# Ventral onlay glanuloplasty for treatment of fossa navicularis strictures

**DOI:** 10.1590/S1677-5538.IBJU.2022.0067

**Published:** 2022-07-10

**Authors:** George Wayne, Alejandra Perez, Timothy Demus, Adam Nolte, Chase Mallory, Jessica Boyer, Billy Cordon

**Affiliations:** 1 Columbia University at Mount Sinai Medical Center Division of Urology Miami Beach Fl USA Division of Urology, Columbia University at Mount Sinai Medical Center, Miami Beach, Fl, USA;; 2 Florida International University School of Medicine Fl USA Florida International University School of Medicine, Fl, USA

**Keywords:** Urethral Stricture, Mouth Mucosa

## Abstract

**Purpose:**

Management of fossa navicularis (FN) strictures balances restoring urethral patency with adequate cosmesis. Historically, FN strictures are managed via glans cap or glans wings, and in severe cases, multi-stage procedures. Ventral onlay glanuloplasty (VOG) is an easily reproducible technique that involves a single-stage augmentation with buccal mucosal graft. We have been applying this technique for several years and present early promising outcomes of this novel approach.

**Materials and Methods:**

We retrospectively reviewed all patients with FN strictures who underwent VOG at our institution. Treatment success was designated by the absence of extravasation on voiding cystourethrogram and no need for further urethral instrumentation on follow up. Glans cosmesis was assessed by patients providing binary (yes/no) response to the satisfaction in their appearance. We also noted stricture length, stricture etiology, demographic characteristics and any post-operative complications and reported median, interquartile range (IQR) and count, frequency (%), accordingly.

**Results:**

Ten patients underwent VOG and fit our inclusion criteria. Median stricture length was 2.0 cm (IQR 1.6 -2). Success rate was 90% (9/10) with a median follow up of 30 months (IQR 24.3 – 36.8). The one recurrence was treated by dilation combined with triamcinolone injection at 419 days post-op. Stricture etiology included primarily iatrogenic causes such as transurethral prostate resection (4/10), greenlight laser vaporization (2/10), cystolitholapaxy (1/10), and traumatic catheterization (3/10). All patients were satisfied with penile cosmesis.

**Conclusion:**

VOG is a simple technique for treating FN strictures. Based on our preliminary series, VOG provides sustained distal urethral patency and patients are pleased with the appearance.

## INTRODUCTION

Fossa navicularis (FN) strictures comprise 18% of all anterior urethral strictures (1). The most described etiology of FN stricture is lichen sclerosis (LS), a chronic inflammatory dermatosis affecting the glans, prepuce and urethra (2-4). Other common causes include idiopathic, iatrogenic, trauma, or prior hypospadias repair (2-4). Despite being a relatively common occurrence, the precise management of FN strictures remains unclear. The inherent difficulty of repair lies in restoring urethral patency while maintaining adequate penile cosmesis (5-7). Ultimately, the chosen approach tailors to patient-specific factors such as the length of stricture, extent of surrounding spongiofibrosis, quality of distal penile skin and presence of LS (6, 8). Approaches to management range from less invasive options such as endoscopic dilation, direct vision internal urethrotomy and extended meatotomy to more extensive reconstructions such as flap and graft urethroplasty. Ventral onlay graft placement, or ventral onlay glanuloplasty (VOG), for FN strictures is a straightforward approach with limited available studies in the literature. We hypothesize that the outcomes of VOG have success rates similar to other surgical techniques in repairing the FN. The aim of this study is to describe our early experience with VOG using buccal mucosa, and to contextualize this technique in the broader literature on FN urethral reconstruction.

## MATERIALS AND METHODS

### Data Collection and Analysis

All urethroplasty cases at our institution performed by a single reconstructive urologist from January 2018 to June 2021 were compiled in an institutional IRB approved database which we used to obtain patient data for this study. This study was approved by our institution’s IRB (FWA00000176).

### Inclusion/Exclusion Criteria

Only single stage FN repairs with VOG were included. Our inclusion criteria consisted of patients with isolated FN strictures that did not extend continuously to more proximal segments of the urethral. We did however include patients with skip lesions in the bulbar or membranous urethra, but focused on their FN repair for the purpose of this study. We excluded patients with lichen sclerosis and excluded FN repairs that were not performed as a VOG as well as any multi-stage repairs. Additionally, for the purposes of this study, VOG repair with adjunctive maneuvers, ie an additional dorsal inlay, were excluded to maintain a homogenous treatment group.

### Outcome Measures

We defined treatment success as (1) the absence of extravasation on VCUG at 3 weeks and (2) no need for urethral dilation or other instrumentation. We also noted any 30-day complications. Stricture etiology, stricture length (cm), prior instrumentation and prior urethroplasty were also noted as well as background patient data such as age, ethnicity, BMI, hypertension, diabetes, and smoking history. We routinely assess penile cosmesis by asking patients whether they were satisfied subjectively with the appearance of their penis (binary: yes/no response) during subsequent follow up visits. This was recorded and reported. Follow up time was reported in months. Measures were reported as count, frequency (%) or median (interquartile range (IQR)), accordingly.

## SURGICAL TECHNIQUE

### Ventral Onlay Glanuloplasty

**Figure 1:**
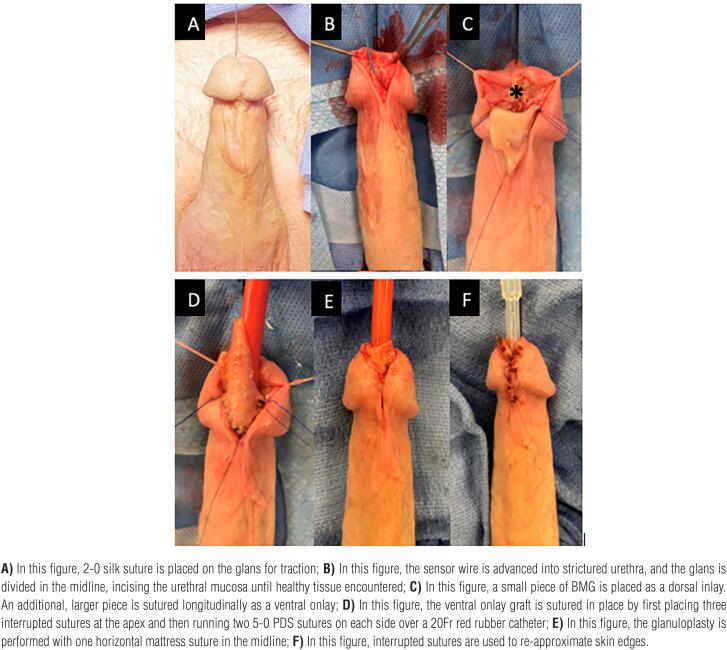
Ventral Onlay Glanuloplasty Technique.

**Figure 2:**
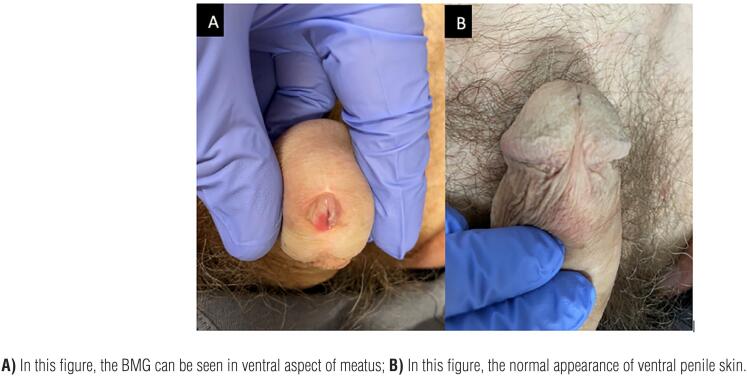
One-year follow up after VOG.

With the patient in supine position, a 2-0 silk suture is placed on the glans for traction and a sensor wire is advanced through the lumen of the strictured urethra, if possible. A ventral incision is made directly through the glans in the midline starting at the meatus, extending proximally while incising urethra until healthy mucosa is identified. Stay sutures with 4-0 vicryl are placed on the edges of the urethral mucosa for traction, and a cystoscopy is performed to rule out any additional areas of narrowing. A BMG is then harvested and defatted in the standard fashion. (At this time, if the narrowing is particularly severe, a dorsal “Asopa” inlay may be used for further augmentation). For the ventral onlay, the BMG is anastomosed in a longitudinal fashion with interrupted sutures at the apex and a running 5-0 PDS suture is used for the lateral edges over a 20Fr red rubber catheter. After BMG anastomosis is complete, a flap of Dartos fascia is approximated over the repair and the glans is reapproximated for further support of the graft. Of note, we do not undermine the glans in order to create glans wings for closure, but rather reapproximate the edges of the glans with an initial deep horizontal mattress suture, followed by interrupted sutures. We create the neomeatus by suturing the distal edge of the graft to the glans itself, calibrating over a 20Fr red rubber catheter. Patients are discharged with a Foley catheter that is removed during the first follow up visit at 3 weeks where urethral patency is assessed with VCUG. Routine follow up then continues at 1 month, 3 months and annually thereafter. Surgical technique and follow up images can be found in Figures 1 and 2.

## RESULTS

We reviewed our institutional database which consisted of 151 urethroplasties performed by a single surgeon. We identified 17 urethroplasties involving FN stricture repair, 11 of which used the VOG surgical technique. One patient was excluded due to lichen sclerosis stricture etiology. Thus, 10 patients were included in total.

Median age was 65.5 years (IQR: 60-69.8) and median body mass index was 25.2 (IQR: 23.1-29.4). Ethnicity breakdown included 40% white Hispanic, 40% white non-Hispanic and 20% Asian. 50% of patients were diabetic, 90% had hypertension and 40% had a smoking history.

Eighty percent of patients had a stricture etiology related to a prior surgery for BPH: 1/7 had cystolitholapaxy, 2/7 underwent greenlight laser vaporization and 5/7 underwent bipolar transurethral resection of the prostate (TURP). The remaining 2 patients had a stricture secondary to traumatic catheterization. Four patients had a prior urethral dilation and one patient had a direct vision internal urethrotomy (DVIU).

Median measured fossa stricture length was 2 cm (IQR: 1.6 – 2.0). The overall success rate was 90% with a median follow up to date of 30 months (IQR: 24.3 – 36.8). One patient developed a recurrence at 14.0 months and was managed by urethral dilation with triamcinolone injection. Since re-intervention, this patient reports strong urinary stream without spray and complete emptying. There were no complications in the study (Table-1).

**Table 1:** Patient background and outcomes.

ID	Age	BMI	Race	DM	HTN	Smoker	Stricture Etiology	Prior Dilation Or DVIU	Defect Length (cm)	Success	Time to Failure (days)
1	71	20	WNH	N	Y	Never	TURP	Dilation	1.5	yes	-
2	71	23	WH	Y	Y	Former	TURP	None	2	yes	-
3	66	24	WNH	N	Y	Never	Greenlight	None	2	yes	-
4	59	26	WNH	N	Y	Former	Catheter	Dilation	3	no*	419
5	66	25	WH	N	N	Never	Other‡	Dilation	2	yes	-
6	54	31	WH	N	Y	Never	TURP	None	2	yes	-
7	31	31	A	Y	Y	Former	Catheter	DVIU	1.5	yes	-
8	63	25	WH	Y	Y	Current	TURP	None	2	yes	-
9	65	31	WNH	Y	Y	Never	Greenlight	None	2.5	yes	-
10	73	22	A	Y	Y	Never	Catheter	Dilation	1.5	yes	-

**Abbreviations:** BMI = Body Mass Index, DM = diabetes mellitus, HTN = hypertension, DVIU = direct vision internal urethrotomy, WNH = White non-Hispanic, WH = White Hispanic, A = Asian, TURP = Transurethral resection of prostate, Greenlight = greenlight laser vaporization, Catheter = catheterization,

**Other‡:** = cystolitholapaxy

* patient had decreased flow on follow up and was treated by dilation with triamcinolone injection

On routine follow up visits, patients were asked about their satisfaction regarding penile cosmesis. All patients reported satisfaction with the appearance of their reconstruction.

## DISCUSSION

The challenge in treatment of FN strictures lies in the dichotomy of maintaining a cosmetically appealing glans and achieving satisfactory functional outcomes. Patient-specific factors and surgeon preference play a role in choosing between the range of available repairs. Patients with LS, hypospadias and those with completely obliterated strictures pose an even greater challenge and traditionally require a complex multi-stage approach, which albeit successful, can be psychologically and physically taxing to the patient. Single-stage repairs have been popularized as they offer high rates of success in one anesthetic session with good aesthetic outcomes. Some still employ ventral flap repairs, first described by Orandi, with either closure with glans wings or utilizing the more labor-intensive glans cap to avoid dividing the glans. Single-stage BMG urethroplasty is particularly promising in treatment of FN strictures of any etiology as the glans provides a robust vascular bed for graft take. However, much of the literature thus far has described dorsal placement of the BMG partly due to concern for fistula formation. Many series have described techniques to prevent fistula formation in anterior urethroplasties such as overlying repair with a tunica vaginalis flap as described by Favorito and colleagues. (9). However, we believe that for isolated FN strictures, particularly in the setting of iatrogenic etiologies, there is a good graft take and low rate of urethrocutaneous fistula without need for interposition flaps.

Our preliminary series of 10 patients provides evidence in favor of VOG as a surgical technique for FN strictures. Surgically, we feel that the procedure has a short learning curve, with short operative time and favorable long-term outcomes. Despite our results being limited to a small sample size, they echo similar series in the literature and attest to the efficacy of VOG in the glans urethra. Although the majority of FN strictures in our series resulted from an iatrogenic cause, we believe there is a role for single-stage repair with VOG in strictures of any etiology, including milder cases of LS. However, in those with severe disease with completely obliterated lumen, a two-stage repair would still be the treatment of choice.

Early approaches to FN reconstruction focused on using vascularized skin flaps to minimize graft-take issues. In 1987, Jordan first described transposing a ventral skin island flap with a vascularized dartos pedicle onto a distal urethrotomy defect and closing with glans wings (6). The surgeon went on to report an 83% success rate with this technique over a mean follow-up of 10.2 years (N= 35). They found success in 23/23 patients without LS and recurrence in 6/12 patients with LS (10). Like Jordan, Armenakas et al. used a ventral transverse island flap but approached and closed the glans differently. Through a subcoronal incision, they dissected under the glans to create a glans cap to access the FN. This method precluded closing with glans wings, which may reduce scarring and fistulization as well as improve cosmesis. In their study, 1 recurrence occurred in 15 patients that received glans cap over a mean follow up of 42 months (11). The cosmetic advantage of performing a glans cap has not been established, since Fiala et al. reported 100% satisfaction with appearance following FN repair that utilized glans wings (N=21) (12).

Moreover, grafted FN repairs offer advantages, especially in inflammatory strictures where the use of diseased genital skin increases failure rate (2, 10). In 28 patients with lichen sclerosis, Venn and Mundy compared genital skin flaps to two-staged grafted repairs utilizing non-genital skin (either full-thickness postauricular skin or buccal mucosa). One of 16 patients in the non-genital skin group had recurrence while 12 of 12 patients in the genital skin flap group had a recurrence, concluding that penile skin flaps should not be used to repair urethral stricture caused by LS (13). In this vein, Gelman et al. combined Jordan’s preputial flap with a dorsal inlay of buccal mucosa to repair FN strictures in one stage. All 12 patients were stricture-free with a mean follow up of 39 months; however, 2 patients developed a urethral fistula (14).

To our knowledge, only two studies have described ventral onlay of BMG for FN strictures (14). In their series, Chowdhury et al. reported 5 out of 6 patients (83%) to have durable functional and cosmetic outcomes at median follow up of 37 months (20). However, we believe our approach has key differences that make it simpler. For example, we do not raise glans wings, which decreases dissection and bleeding. We also do not take anchoring bites on the graft during glans apposition. After graft placement and closure of dartos, we approximate the glans to relieve tension with a deep horizontal mattress suture using 2-0 monocryl, bringing the edges together in a natural position without overly compressing the graft. We then re-approximate the skin edges in an interrupted fashion and suture the distal edge of BMG to the glans itself to create the neomeatus. Conversely, Chowdhury describes suturing the distal edge of the BMG to the margins of the initial ventral slit made on the meatus. Goel et al. expanded use of BMG to both a dorsal inlay and ventral onlay, reporting ‘satisfactory clinical outcomes’ in 10 of 10 patients at a mean follow up of 13.5 months (15).

Given the heterogeneity in effective treatment approaches, one group recently proposed an algorithm based on stricture etiology and functional anatomy (16). They demonstrated that single-stage repair with dorsal inlay BMG is suitable for most FN strictures but recommend a multistage approach for hypospadias and those with severe LS (1). They use ventral fasciocutaneous flap as an adjunct if urethra cannot be calibrated to 20Fr after dorsal inlay. Although much of the literature describes dorsal placement of BMG, our series supports that ventral BMG onlay (± dorsal inlay) can be a valuable addition to the FN reconstruction armamentarium.

Recent innovations explore the use of minimally invasive modalities of FN stricture repair without skin incision. Nikolavsky et al. first described a transurethral placement of BMG as an inlay following wedge resection of the narrowed segment. They reported no recurrences, no fistula formations, and improved flow in 3 out of 3 patients with at least 1 year follow up (range 12-24 months) (17). The procedure was studied again but at a multi-institutional level (15 sites total), finding 3 recurrences in 57 patients with a median follow up of 17 months (IQR 13-22) (17). Others have even successfully adapted transurethral circular graft placement through a circum-meatal incision and careful dissection of the urethra from the glans. Notably, 9 out of 19 patients required proximal ventral urethrotomy due to inadequate urethral exposure (19). These approaches may offer a feasible single-stage option for distal FN stricture repair with avoidance of skin incision or urethral mobilization. However, they may prove more surgically challenging, particularly in longer and more proximal FN strictures.

Our study had a small sample size of 10 patients, however, it was larger than the only other study presenting VOG for FN strictures, which included 6 patients in total (20). Furthermore, the majority of patients in that analysis had lichen sclerosis stricture etiology whereas the vast majority of strictures in our study were iatrogenic. Thus, our findings show the feasibility of VOG for iatrogenic FN strictures. Another limitation involved our definition of treatment success with no further need for urethral instrumentation. While objective measures are limited by patient follow up, this was the only practice measure from our database that has been used as an outcome measure in prior published series (10, 15, 18). In addition, cosmetic satisfaction as a binary question is limited, however, this measure has been used in other series as well (12, 15, 16). Due to the retrospective nature of the study, we were unable to provide validated patient reported outcomes on penile or glans cosmesis. Despite this, our preliminary data provides evidence for the feasibility of ventral onlay for fossa navicularis strictures and adds to the armamentarium of urethral reconstructive techniques.

## CONCLUSION

A variety of techniques are available for addressing glans urethral strictures. This study has the potential to further change clinical practice as ventral grafts have long been used in the bulbar urethra but not as commonly in the distal urethra. VOG shows promise to treat various stricture etiologies providing patency and pleasing cosmesis. Given the short learning curve of this technique and the promising early results, we feel that VOG belongs in most reconstructive urologists’ toolkits for addressing the often-challenging case of the FN stricture.

## ABBREVIATIONS

FN: Fossa Navicularis
VOG: Ventral Onlay Glanuloplasty
BMG: Buccal Mucosal Graft
LS: Lichen Sclerosis
VCUG: Voiding Cystourethrogram

## References

[1] Fenton AS, Morey AF, Aviles R, Garcia CR. Anterior urethral strictures: etiology and characteristics. Urology. 2005;65:1055-8. 10.1016/j.urology.2004.12.018 15913734

[2] Dielubanza EJ, Han JS, Gonzalez CM. Distal urethroplasty for fossa navicularis and meatal strictures. Transl Androl Urol. 2014;3:163-9. 10.3978/j.issn.2223-4683.2014.04.02 PMC4708167 26816765

[3] Palminteri E, Berdondini E, Verze P, De Nunzio C, Vitarelli A, Carmignani L. Contemporary urethral stricture characteristics in the developed world. Urology. 2013;81:191-6. 10.1016/j.urology.2012.08.062 23153951

[4] Stein DM, Thum DJ, Barbagli G, Kulkarni S, Sansalone S, Pardeshi A, et al. A geographic analysis of male urethral stricture aetiology and location. BJU Int. 2013;112:830-4. 10.1111/j.1464-410X.2012.11600.x 23253867

[5] Daneshvar M, Hughes M, Nikolavsky D. Surgical Management of Fossa Navicularis and Distal Urethral Strictures. Curr Urol Rep. 2018;19:43. 10.1007/s11934-018-0792-1 29667080

[6] Jordan GH. Reconstruction of the fossa navicularis. J Urol. 1987;138:102-4. 10.1016/s0022-5347(17)43006-0 3599186

[7] Babu P, Nayak A, Javali TD, Joshi P, Nagaraj HK, Aggarwal K. Evaluation of Jordan’s meatoplasty for the treatment of fossa navicularis strictures. A retrospective study. Cent European J Urol. 2017;70:103-6. 10.5173/ceju.2017.916 PMC5407332 28461997

[8] Singh SK, Agrawal SK, Mavuduru RS. Management of the stricture of fossa navicularis and pendulous urethral strictures. Indian J Urol. 2011;27:371-7. 10.4103/0970-1591.85442 PMC3193739 22022062

[9] Favorito LA, da Silva FS Filho, de Resende JA Junior. A new option to prevent fistulas in anterior urethroplasty in patients with kippered urethra: the tunica vaginalis flap. Int Braz J Urol. 2021;47:1032-6. 10.1590/S1677-5538.IBJU.2020.1058 PMC8321442 34260180

[10] Virasoro R, Eltahawy EA, Jordan GH. Long-term follow-up for reconstruction of strictures of the fossa navicularis with a single technique. BJU Int. 2007;100:1143-5. 10.1111/j.1464-410X.2007.07078.x 17627782

[11] Armenakas NA, Morey AF, McAninch JW. Reconstruction of resistant strictures of the fossa navicularis and meatus. J Urol. 1998;160:359-63. 9679877

[12] Fiala R, Vrtal R, Zenisek J, Grimes S. Ventral prepucial flap meatoplasty in the treatment of distal urethral male strictures. Eur Urol. 2003;43:686-8. 10.1016/s0302-2838(03)00186-6 12767371

[13] Venn SN, Mundy AR. Urethroplasty for balanitis xerotica obliterans. Br J Urol. 1998;81:735-7. 10.1046/j.1464-410x.1998.00634.x 9634051

[14] Gelman J, Sohn W. 1-stage repair of obliterative distal urethral strictures with buccal graft urethral plate reconstruction and simultaneous onlay penile skin flap. J Urol. 2011;186:935-8. 10.1016/j.juro.2011.04.056 21791347

[15] Goel A, Goel A, Dalela D, Sankhwar SN. Meatoplasty using double buccal mucosal graft technique. Int Urol Nephrol. 2009;41:885-7. 10.1007/s11255-009-9555-8 19350407

[16] Broadwin M, Vanni AJ. Outcomes of a urethroplasty algorithm for fossa navicularis strictures. Can J Urol. 2018;25:9591-5. 30553284

[17] Nikolavsky D, Abouelleil M, Daneshvar M. Transurethral ventral buccal mucosa graft inlay urethroplasty for reconstruction of fossa navicularis and distal urethral strictures: surgical technique and preliminary results. Int Urol Nephrol. 2016;48:1823-9. 10.1007/s11255-016-1381-1 27470030

[18] Daneshvar M, Simhan J, Blakely S, Angulo JC, Lucas J, Hunter C, et al. Transurethral ventral buccal mucosa graft inlay for treatment of distal urethral strictures: international multi-institutional experience. World J Urol. 2020;38:2601-7. 10.1007/s00345-019-03061-6 31894369

[19] Onol SY, Onol FF, Gümüş E, Topaktaş R, Erdem MR. Reconstruction of distal urethral strictures confined to the glans with circular buccal mucosa graft. Urology. 2012;79:1158-62. 10.1016/j.urology.2012.01.046 22449449

[20] Chowdhury PS, Nayak P, Mallick S, Gurumurthy S, David D, Mossadeq A. Single stage ventral onlay buccal mucosal graft urethroplasty for navicular fossa strictures. Indian J Urol. 2014;30:17-22. 10.4103/0970-1591.124200 PMC3897046 24497676

